# SARS-CoV-2 spike produced in insect cells elicits high neutralization titres in non-human primates

**DOI:** 10.1080/22221751.2020.1821583

**Published:** 2020-09-24

**Authors:** Tingting Li, Qingbing Zheng, Hai Yu, Dinghui Wu, Wenhui Xue, Hualong Xiong, Xiaofen Huang, Meifeng Nie, Mingxi Yue, Rui Rong, Sibo Zhang, Yuyun Zhang, Yangtao Wu, Shaojuan Wang, Zhenghui Zha, Tingting Chen, Tingting Deng, Yingbin Wang, Tianying Zhang, Yixin Chen, Quan Yuan, Qinjian Zhao, Jun Zhang, Ying Gu, Shaowei Li, Ningshao Xia

**Affiliations:** a State Key Laboratory of Molecular Vaccinology and Molecular Diagnostics, School of Life Sciences, School of Public Health, Xiamen University, Xiamen, People’s Republic of China; b National Institute of Diagnostics and Vaccine Development in Infectious Disease, Xiamen University, Xiamen, People’s Republic of China; c Department of Pulmonary Medicine, The First Affiliated Hospital of Xiamen University, Xiamen, People’s Republic of China; d The Research Unit of Frontier Technology of Structural Vaccinology of Chinese Academy of Medical Sciences, People’s Republic of China

**Keywords:** COVID-19, SARS-CoV-2, spike, insect cell expression system, immunogenicity

## Abstract

The current coronavirus disease 2019 (COVID-19) pandemic was the result of the rapid transmission of a highly pathogenic coronavirus, severe acute respiratory syndrome coronavirus 2 (SARS-CoV-2), for which there is no efficacious vaccine or therapeutic. Toward the development of a vaccine, here we expressed and evaluated as potential candidates four versions of the spike (S) protein using an insect cell expression system: receptor binding domain (RBD), S1 subunit, the wild-type S ectodomain (S-WT), and the prefusion trimer-stabilized form (S-2P). We showed that RBD appears as a monomer in solution, whereas S1, S-WT, and S-2P associate as homotrimers with substantial glycosylation. Cryo-electron microscopy analyses suggested that S-2P assumes an identical trimer conformation as the similarly engineered S protein expressed in 293 mammalian cells but with reduced glycosylation. Overall, the four proteins confer excellent antigenicity with convalescent COVID-19 patient sera in enzyme-linked immunosorbent assay (ELISA), yet show distinct reactivities in immunoblotting. RBD, S-WT and S-2P, but not S1, induce high neutralization titres (>3-log) in mice after a three-round immunization regimen. The high immunogenicity of S-2P could be maintained at the lowest dose (1 μg) with the inclusion of an aluminium adjuvant. Higher doses (20 μg) of S-2P can elicit high neutralization titres in non-human primates that exceed 40-times the mean titres measured in convalescent COVID-19 subjects. Our results suggest that the prefusion trimer-stabilized SARS-CoV-2 S-protein from insect cells may offer a potential candidate strategy for the development of a recombinant COVID-19 vaccine.

## Introduction

Severe acute respiratory syndrome coronavirus 2 (SARS-CoV-2) is now the third highly pathogenic virus from the betacoronavirus genus to causes serious illness and death in humans following the epidemics caused by severe acute respiratory syndrome coronavirus (SARS-CoV) in 2002 and Middle East respiratory syndrome coronavirus (MERS-CoV) in 2012 [1]. In phylogenic terms, SARS-CoV-2 is genetically close to bat coronavirus and SARS-CoV [2]. The SARS-CoV-2 causative disease “Coronavirus disease 2019 (COVID-19),” which first emerged in late 2019 in the Hubei Province of China, is characterized by high fever, dry cough, difficulty breathing and severe atypical pneumonia, with infection confirmed by a positive virus RNA test or pulmonary computed tomography (CT) in clinical practice [3,4]. SARS-CoV-2 has spread to over 200 countries, and resulted in over 7.4 million confirmed cases worldwide with approximately 41,800 deaths and counting (https://www.who.int). Because of the much higher human-to-human transmissibility than that exhibited by the other coronavirus outbreaks [5], the World Health Organization (WHO) declared SARS-CoV-2 a pandemic of international concern.

SARS-CoV-2 is an enveloped, single-stranded, positive-sense RNA virus, with a genome of ∼30 kb. There are at least three membrane proteins: a surface spike protein (S), an integral membrane protein (M), and an envelope protein (E). Like other coronaviruses, the S protein is a homotrimeric glycoprotein and is responsible for engaging angiotensin-converting enzyme 2 (ACE2) receptor and mediating cell-virus membrane fusion by the class I fusion mechanism [6,7]. Thus, the S protein is the main target for neutralizing antibodies against viral infection and therefore is usually the core immunogen constituent of vaccine design. The S protein is composed of the S1 and S2 subunits, with cleavage on the S1/S2 boundary by proteases during biosynthesis a prerequisite for coronavirus cellular membrane fusion and subsequent infection [8]. Unlike SARS-CoV and other bat coronaviruses, SARS-CoV-2 evolved a 4-residue insertion (RRAR) at positions 682–685 as a potential furin cleavage site, which may have contributed to the higher transmissibility of this novel coronavirus [8,9]. This S protein is metastable and is present in two conformational states: prefusion and post-fusion states. Previous studies have suggested that the infection process of MERS-CoV [10] and SARS-CoV [11] involves a conformational transition of the S trimer from a prefusion conformation ready for ACE2 binding to a post-fusion conformation for eventual virus-cell membrane fusion. There are several cryo-electron microscopy (cryo-EM) structures of SARS-CoV-2 spike trimers in the prefusion conformation [9,12]. Vaccines based on prefusion states mostly preserve the initially native contours of the viral spike and thus elicit high-quality neutralizing antibodies against virus infection.

In enveloped viruses, such as human immunodeficient virus and influenza virus, glycans linked to the surface spike protein play extensive roles in the virus life cycle, including virus-host recognition and immune evasion. However, these glycans also raise major challenges for vaccine development, as they can shield some epitopes from being accessed by immune effectors in association with the elicitation of neutralizing antibodies [13–16]. Indeed, the spike protein of SARS-CoV-2 has dense glycosylation when expressed in mammalian cells; the underlying glycosylation sites have been characterized by cryo-EM and liquid-chromatography-mass spectrometry (LC-MS) [17]. However, whether S-protein glycosylation noted in other systems is related to the immunogenicity of potent neutralizing antibodies and the protection efficacy of recombinant vaccines remains unclear.

Emerging immunogenicity and efficacy data from non-human primates have been presented using an inactivated [18] or an S-based DNA [19] vaccine candidate, in which the S proteins served as the major immunogen component are essentially expressed in mammalian cells. However, there is still no appropriate vaccine or drug for COVID-19 or the prevention of SARS-CoV-2 infection. Indeed, one that considers prefusion states and glycosylation extent is warranted. To this end, in the present study, we cloned the S-protein ectodomain in wild-type and trimer-stabilized forms in prefusion state, as well as the S-protein fragments, RBD and S1, into a recombinant baculovirus and expressed these four proteins in insect cells. Using cryo-EM, glycosylation, antigenicity and immunogenicity analyses, we explored whether a SARS-CoV-2 S-protein trimer could be a potent immunogen for applications in recombinant vaccine development for SARS-CoV-2.

## Materials and methods

### Cloning, protein expression and purification

The SARS-CoV-2 S gene (Genbank accession no. NC_045512.2) was synthesized and cloned into a baculovirus shuttle vector pAcgp67B (BD Biosciences, CA, USA) using Gibson assembly. The S-WT construct was cloned from the gene of SARS-CoV-2 S ectodomain encoding amino acids (aa) 15-1,213. The original signal sequence was removed and the product cloned downstream of the gp67 signal sequence in the pAcgp67B plasmid vector, along with a C-terminal thrombin cleavage site, a T4 trimerization foldon motif, and a His tag. The trimer-stabilized S-2P has an identical configuration to the S-WT but with two proline substitutions at residues 986 and 987 and the inclusion of an “AGAG” at the furin cleavage site (residues 682-685). The segments S1 (aa 15-680) and RBD (aa 319-541) were cloned in a manner similar to the S ectodomain, maintaining the gp67 signal peptide and His tag to facilitate secretion outside the cell and affinity purification, respectively, but without the thrombin site or the T4 foldon.

Protein expression and purification were performed as described previously [20]. All plasmids were co-transfected with linearized 2.0 DNA (deficient in *v-cath/chiA*genes) (Expression Systems, CA, USA) into *Sf9* insect cells (Thermo Fisher Scientific, MA, USA), according to the protocol provided by the manufacturer (Expression Systems). The transfection supernatant was harvested and amplified twice to obtain a high titre of recombinant virus. Hive Five cells (BTI-TN-5B1-4) (Thermo Fisher Scientific) were cultured in ESF921 medium (Expression Systems) and infected with recombinant virus at a multiplicity of infection (MOI) of 5 in the exponential growth phase (2 × 10^6^ cells/ml; 95% viability) at 28°C for 72 h. The culture media was centrifugated at 8,000 rpm for 20 min. The supernatant was dialyzed against phosphate-buffered saline (PBS), pH 7.4, purified with Ni-sepharose fast-flow 6 resin (GE Healthcare, Boston, USA), and eluted with 250 mM imidazole. The protein concentrations of the final purified samples were measured with Pierce BCA Protein Assay Kit (Thermo Fisher Scientific).

### SDS-PAGE and western blot

Equal amounts of protein samples were mixed with loading buffer, boiled for 10 min, and loaded onto two SDS-PAGE gels: one for western blotting and one for Coomassie blue staining, following standard laboratory protocols. Proteins were electrophoresed for 70 min at 80 V in a BioRad MINI-PROTEAN Tetra system (BioRad Laboratories, CA, USA), and the gel was stained with Coomassie Brilliant Blue R-250 (Bio-Rad) for 30 min at room temperature. For western blotting, separated proteins were transferred onto a nitrocellulose membrane (Whatman, Dassel, Germany) using a Trans-Blot Turbo transfer system (Bio-Rad). The membrane was blocked and then incubated for 1 h with an His-tag-specific mouse mAb antibody (Proteintech, Rosemont, USA) or human sera (1:500 dilution). Unbound antibody was removed by five 5-min washes and the membrane was incubated with alkaline phosphatase-conjugated goat anti-mouse secondary antibody or goat anti-human IgG secondary antibody (Abcam, Cambridge, UK). Membranes were washed again and then developed using SuperSignal ELISA Pico Chemiluminescent Substrate Kit (Thermo Fisher Scientific).

### Enzyme-linked immunosorbent assay (ELISA)

Purified proteins were coated into the wells of 96-well microtitre plates at 100 ng/well in PBS and incubated at 37°C for 4 h. The background was blocked with 1 × enzyme dilution buffer (PBS + 0.25% casein + 1% gelatin + 0.05% proclin-300) at 37°C for 2 h. Sera at 1:100 were three-fold serially diluted, added to the wells (100 µl), and incubated at 37°C for 1 h. A horseradish peroxidase (HRP)-labeled mouse anti-human antibody (Abcam) was used as the secondary antibody at 1:5,000 for 30 min. Wells were washed again and the reaction catalyzed using o-phenylenediamine (OPD) substrate at 37°C for 10 min. The OD_450nm_ (reference, OD_620nm_) was measured on a microplate reader (TECAN, Männedorf, Switzerland) with a cut-off value of 0.1. The half-effective titres (ET_50_) were calculated by sigmoid trend fitting using GraphPad Prism software (GraphPad Software, CA, USG).

### Size-exclusive chromatography (SEC)

Ni-NTA purified S proteins were further purified using Superdex 200 columns (GE Healthcare). The fractions were harvested and analyzed by SDS-PAGE. All high-purity RBD, S1, and S proteins were subjected to HPLC (Waters; Milford, MA) analysis using a TSK Gel G5000PWXL7.8 × 300 mm column (TOSOH, Tokyo, Japan) equilibrated in PBS, pH 7.4. The system flow rate was maintained at 0.5 mL/min and eluted proteins were detected at 280 nm.

### Analytical ultracentrifuge (AUC)

The AUC assay was performed using a Beckman XL-Analytical ultracentrifuge (Beckman Coulter, Fullerton, CA), as described elsewhere [21]. The sedimentation velocity (SV) was carried out at 20°C with diluted proteins in PBS. The AN-60 Ti rotor speed was set to 20,000–30,000 rpm according to the molecular weight of the control proteins. Data was collected using SEDFIT computer software, kindly provided by Dr. P. C. Shuck (NIH, Bethesda, MA, USA). Multiple curves were fit to calculate the sedimentation coefficient (S) using the continuous sedimentation coefficient distribution model [c(s)]. The c(s) was then used to estimate protein molar mass.

### Differential scanning calorimetry (DSC)

Differential scanning calorimetry (DSC) was carried out on the S proteins using a MicroCal VP-DSC instrument (GE Healthcare, MicroCal Products Group, Northampton, MA) as described previously [22]. In brief, all samples with a concentration of 0.2 mg/mL were measured at a heating rate of 1.5°C/min with the scan temperature ranging from 10°C to 90°C. The melting temperatures (Tm) were calculated using MicroCal Origin 7.0 (Origin-Lab Corp., Northampton, MA) software assuming a non-two-state unfolding model.

### Endo-H and PNGase-F digestion

Endo-H (NEB, MA, US) and PNGase-F (NEB) digestion reactions were performed according to the manufacturer’s instructions. In brief, deglycosylation reactions were carried out using 10 µg S proteins with 5 µL of Endo H or PNGase F at 37°C overnight. The reactions were separated by SDS-PAGE and analyzed by western blotting using anti-His as the detecting antibody.

### Cryo-EM sample preparation and data collection

Aliquots (3 μL) of 0.5 mg/mL purified S-WT and S-2P proteins incubated in 0.085 mM dodecyl-maltoside (DDM) were loaded onto glow-discharged (60 s at 20 mA) holey carbon Quantifoil grids (R1.2/1.3, 200 mesh, Quantifoil Micro Tools) using a Vitrobot Mark IV (ThermoFisher Scientific) at 100% humidity and 4°C. Data were acquired using the EPU software on an FEI Tecnai F30 transmission electron microscope (ThermoFisher Scientific) operated at 300 kV and equipped with a ThermoFisher Falcon-3 direct detector. Images were recorded in the 58-frame movie mode at a nominal 93,000× magnification with a pixel size of 1.12 Å. The total electron dose was set to 46 e^−^ Å^−2^ and the exposure time was 1.5 s. We collected 537 micrographs with a defocus range of 1.5–2.8 μm.

### Cryo-EM data processing

Movie frame alignment and contrast transfer function estimation of each aligned micrograph were carried out with the programs Motioncor [23] and Gctf [24]. Particles were picked by the “Template picker” session of cryoSPARC v2 [25]. Two rounds of reference-free 2D classification were performed, and well-defined particle images were selected. Non-uniform 3D refinement and 3D reconstruction with C3 symmetry were performed using cryoSPARC v2. The resolutions of the final maps were estimated on the basis of the gold-standard Fourier shell correlation curve with a cutoff at 0.143 [26]. Density-map-based visualization and segmentation were performed with Chimera [27].

### Animal immunization

The experimental protocols were approved by the Xiamen University Laboratory Animal Management Ethics Committee. All manipulations were strictly conducted in compliance with animal ethics guidelines and approved protocols. The mouse group size was 5 per group. Six-week-old BALB/c mice were immunized with an initial intramuscular injection (150 µL) of SARS-CV-2 RBD (100 μg), S1 (35 μg), or S-WT (35 μg) proteins combined with aluminium or 50% Freund’s complete adjuvant (Sigma-Aldrich) into left and right posterior limbs, respectively, at weeks 0, 1, and 4. Dose-dependency was assessed with a separate immunization routine, comparing S-2P and S-WT proteins at 10 μg and 1 μg. Blood samples were collected before each injection and were centrifuged at 13,000 g for 10 min. Serum samples were preserved at −20°C.

To assess the immunogenicity of the S-2P protein in non-human primates, 2 cynomolgus macaques (*Macaca Fascicularis*) – with no detectable neutralizing antibodies against SARS-CoV-2 infection – were inoculated with S-2P (20 μg per dose) three times (weeks 0, 2, 6) by intramuscular injection. Pre-immune serum samples from each monkey were collected individually, and serum samples were collected at 1-week intervals after immunization. The titres of SARS-CoV-2 neutralizing antibodies in these sera were detected by VSV-based pseudovirus-type neutralization assay. Data for the neutralization titres are shown as IC_50_ (dilution fold) and plotted as the mean with SD using Graphpad Prism 8.

### Pseudotype-based neutralization assay

Pseudotype-based neutralization assays were performed as described previously [28]. In brief, culture supernatant of monoclonal hybridoma cells, gradient-diluted purified antibodies, or convalescent serum samples were mixed with diluted VSV-SARS-CoV-2-Sdel18 virus (MOI = 0.05) and incubated at 37°C for 1 h. All samples and viruses were diluted using Dulbecco’s Modified Eagle’s Medium (DMEM) with 10% fetal bovine serum. The mixture was added to seeded BHK21-hACE2 cells and incubated for 12 h. Fluorescence images were obtained using Opera Phenix or Operetta CLS equipment (PerkinElmer). For quantitative determination, fluorescence images were analyzed by Columbus system (PerkinElmer). The numbers of GFP-activated cells for each well were counted to represent infection performance. Neutralizing potency was calculated as the reduction (%) in the number of GFP-activated cells following mAb treatment as compared with the non-treated controls.

## Results

### Construct design, expression and purification of SARS-CoV-2 S proteins

**Figure 1.**
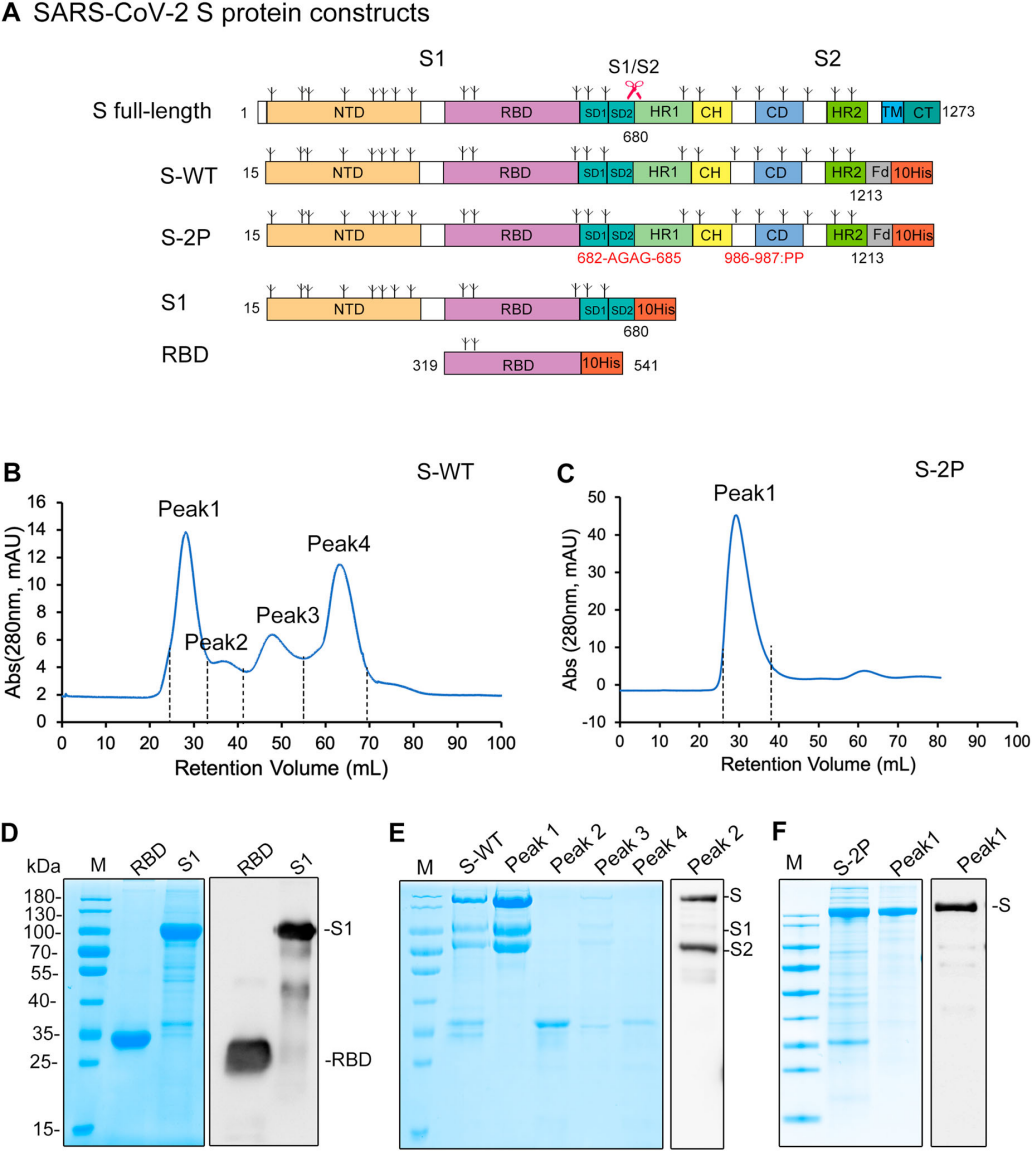
SARS-CoV-2 S protein constructs. (A) Linear representations of the S protein primary structure and construct design. NTD, N-terminal domain; RBD, receptor binding domain; SD1, subdomain 1; SD2, subdomain 2; HR1, heptad repeat 1; CH, central helix; CD, connector domain; HR2, heptad repeat 2; TM, transmembrane domain; CT, cytoplasmic tail; FD, T4 foldon motif. The predicted glycosylation sites are indicated above the domain bars (three-pronged tree symbol). The furin cleavage site located between S1 and S2 is indicated by red scissor. (B, C) Size-exclusion chromatogram (SEC) of the second-step purification of the S-WT and S-2P proteins. (D, E, F) SDS-PAGE and western blotting of the Ni-NTA and SEC purified proteins. RBD and S1 were eluted by 250 mM imidazole, fractions of S-WT and S-2P were harvested from SEC. Anti-His antibody was used as the detection antibody in immunoblotting.

To screen for a potent immunogen for COVID-19 vaccine development, we designed four constructs of the SARS-CoV-2 spike (S) protein: the S-protein ectodomain with the wild-type sequence (S-WT), a previously described trimer stabilization design (S-2P) [29], the S1 subunit, and the RBD fragment. These constructs were expressed by aligning the SARS-CoV-2 S gene (Genbank accession no. NC_045512.2) with a SARS-CoV strain (Genbank accession no. NC_004718) S gene sequence in terms of structure-defined domain profile of the SARS-CoV S protein (Figure 1(A)). To facilitate trimerization of the S proteins, a T4 trimerization foldon was added to the C-terminus of the S-WT and the S-2P constructs with a linkage bearing a thrombin cleavage site for future foldon removal. S-2P was further engineered with mutations, including two proline substitutions at residue 986 and 987, and an “AGAG” replacement at the furin cleavage site (residues 682-685). The four coding genes were cloned downstream of the gp67 signal sequence in the pAcgp67B plasmid vector along with the addition of a His tag. Constructs were co-transfected into Sf9 insect cells with v-cath/chiA gene-deficient baculovirus DNA for the generation and amplification of recombinant baculoviruses. These were then harnessed to infect Hive Five insect cells to produce the recombinant proteins.

The recombinant proteins were mostly solubly expressed and secreted into the culture medium. The centrifugation supernatants of the cell cultures were subjected to metal affinity chromatography using Ni-NTA resin. All four protein types eluted in the separation fractions under 250 mM imidazole elution, and were resolved at molecular weights (m.w.) of ∼170 kDa, 110 kDa, and 35 kDa, respectively, in SDS-PAGE, as detected using an anti-His antibody (Figure 1(D–F)). Interestingly, approximately half of the S-WT proteins were cleaved into S1 (identical migration site to that observed in the S1 lane; Figure 1(D)) and S2 (∼80-kDa, anti-His immunoblot) subunits, possibly by innate furin within the insect cell culture; similar cases of enzymatic cleavage during protein expression have been observed using insect cells, such as Flu HA [30]. The eluted S-WT and S-2P fractions were further polished by Superdex 200 to remove contaminant proteins (Figure 1(B,C)). For S-WT, the peaks, which fractionated at retention volumes of 28 mL, 36 mL, 48 mL, and 65 mL, were subjected to SDS-PAGE analysis. The S proteins, together with the cleaved S1/S2 subunits, were resolved at peak 1 in the size-exclusion chromatography (SEC) (Figure 1(B)) and showed a high purity (>95%) of total S/S1/S2 in the gel (Figure 1(E)). In contrast, there was a single main S-2P peak present in the Superdex 200 chromatography, and the eluted component exclusively resolved as an intact band in SDS-PAGE (Figure 1(F)). The one-step Ni-NTA affinity chromatography produced an RBD fragment of 95% purity (yield, 30 mg/L cell culture), and an S1 protein of ∼90% purity (10 mg/L yield). Further purification through SEC was required for the resultant S samples: S-WT and S-2P had over 95% purity (in the case of S-WT, this was calculated as total S with or without cleavage), with yields of 1 mg/L (S-WT) and 5 mg/L (S-2P) of cell culture, respectively. These yields were the starting point for the further optimization of the SARS-CoV-2 S-immunogen candidates using the insect baculovirus expression vector system (BEVS).

### Physiochemical properties of SARS-CoV-2 S-RBD, S1 and S proteins

**Figure 2.**
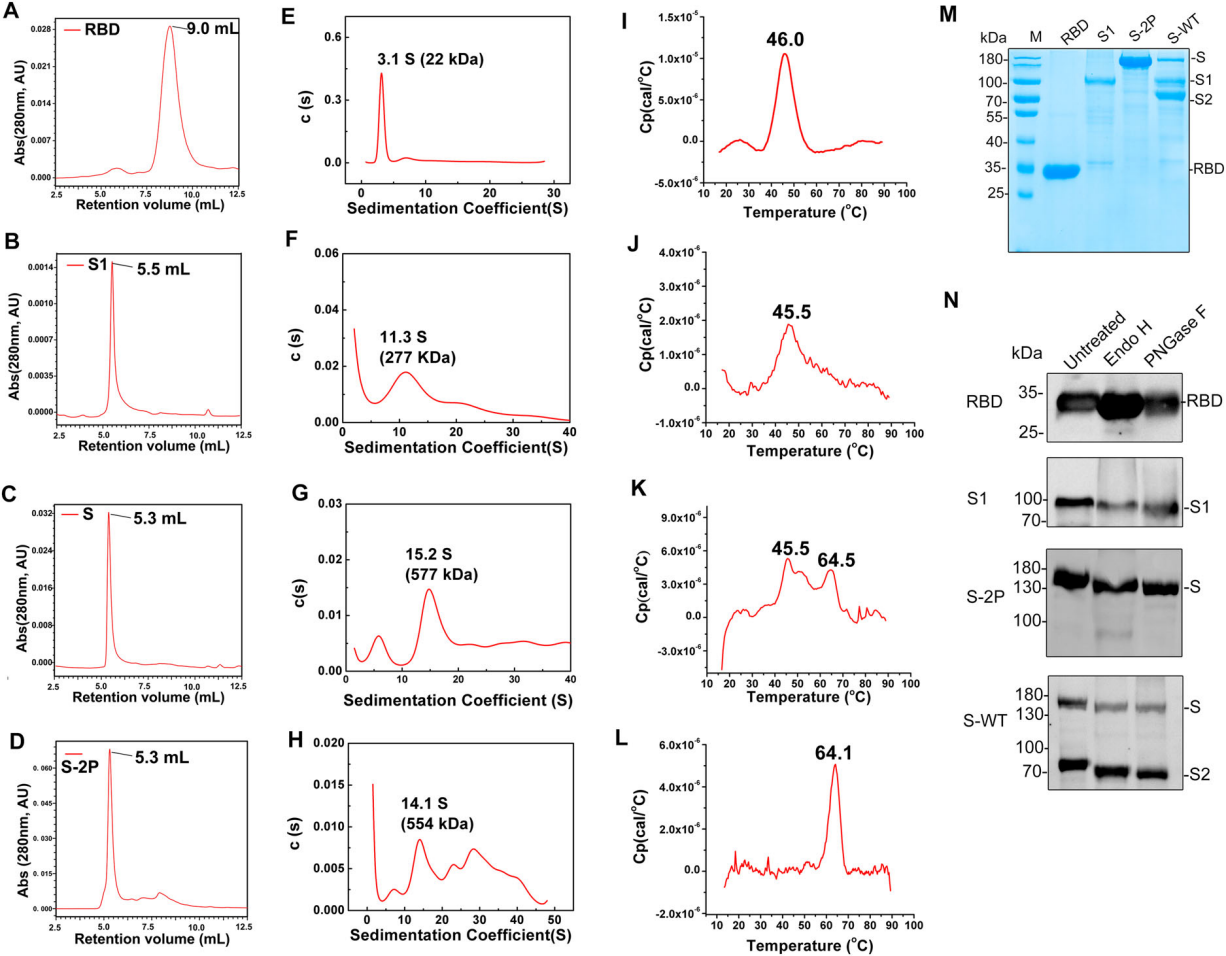
Characterization of the purified RBD, S1, and S proteins. (A-D) High-pressure size-exclusion chromatography (HPSEC) profiles of the purified RBD, S1, S-WT and S-2P proteins. (E-H) Sedimentation velocity analytical ultracentrifugation (AUC) profiles of RBD, S1, S-WT, and S-2P proteins. (I-L) Differential scanning calorimetry (DSC) profiles of RBD, S1, S-WT, and S-2P proteins. (M) Coomassie blue staining of the purified proteins. (N) Immunoblotting of the three purified proteins following treatment with Endo H and PNGase F or untreated as control. Anti-His antibody was used as the detection antibody.

We next investigated the physiochemical properties (association potential, thermal stability, and glycosylation) of the recombinant S proteins from insect cells. First, high-pressure size-exclusion chromatography (HPSEC) and sedimentation velocity analytical ultracentrifugation (SV-AUC) analyses were carried out to measure the oligomerization potential of the four proteins in solution. RBD, S1, S-WT and S-2P all showed a single major peak in the HPSEC profiles at elution volumes of 9.0, 5.5, 5.3, and 5.3 mL, respectively (Figure 2(A–D)). The four proteins were further verified by SV-AUC. RBD sedimented as a single species of 3.1 S in the c(s) profile, corresponding to an apparent molecular weight of 22 kDa (Figure 2(E)). Comparatively, S1 was present as a dominant species of 11.3 S (∼277 kDa, corresponding to an S1 trimer) and a minor aggregate form of 20 S (Figure 2(F)). S-WT resolved at 15.2 S, equivalent to 577 kDa (Figure 2(G)), which is the approximate theoretical molecular weight of an intact S trimer. S-2P showed a main peak of 14.1 S, approximately equivalent to 544 kDa (Figure 2(H)).

The four proteins were further analyzed by differential scanning calorimetry (DSC) to investigate the inner thermostability [22]. RBD and S1 each showed one major peak at comparable thermal denaturation midpoints (Tm) of 46.0 °C and 45.5 °C, respectively (Figure 2(I,J)), whereas S-WT showed two major peaks at Tm of 45.5°C (identical to Tm of S1) and 64.5°C (Figure 2(K)); these values might reflect the coexistence of intact S and cleaved S1/S2. In contrast, S-2P presented as a sharp peak of 64.1°C in the DSC profile (Figure 2(L)), indicating a higher homogeneity and overall better thermostability than S-WT.

Finally, we used enzymatic deglycosylation analysis to investigate the glycosylation extent of the four proteins (Figure 2(M,N)). Samples were treated with either Endo H or PNGase F: (1) Endo H unleashes the chithobiose core of high mannose and some hybrid oligosaccharides from the N-linked glycoproteins, i.e. it removes the extended glycan branches, and leaves behind one N-acetylglucosamine (GlcNAc) on the N-linked glycoproteins [31]; (2) PNGase F, on the other hand, releases N-linked glycan moieties between GlcNAc and ASN residues within a glycoprotein. It should be noted that, in insect cells, glycosylation appears as terminal mannose glycans, unlike complex sialylated glycans in mammalian cells, and that glycosylation correlates with immunogenicity and broad-coverage protection of a glycoprotein immunogen [13,14]. Treatment with either Endo H or PNGase F led to no discernible decrease in the molecular weight of RBD in SDS-PAGE, but nearly a 10-kDa decrease for both S1 and S2, and a ∼20-kDa decrease for both S-WT and S-2P (Figure 2(N)). From these analyses, we can conclude that the extent of glycosylation within the S glycoprotein is RBD < S1 ∼ S2, which is consistent with the predicted glycosylation profile of the S polypeptide (Figure 1(A)).

### Cryo-EM structure of the trimer-stabilized SARS-CoV-2 spike

**Figure 3.**
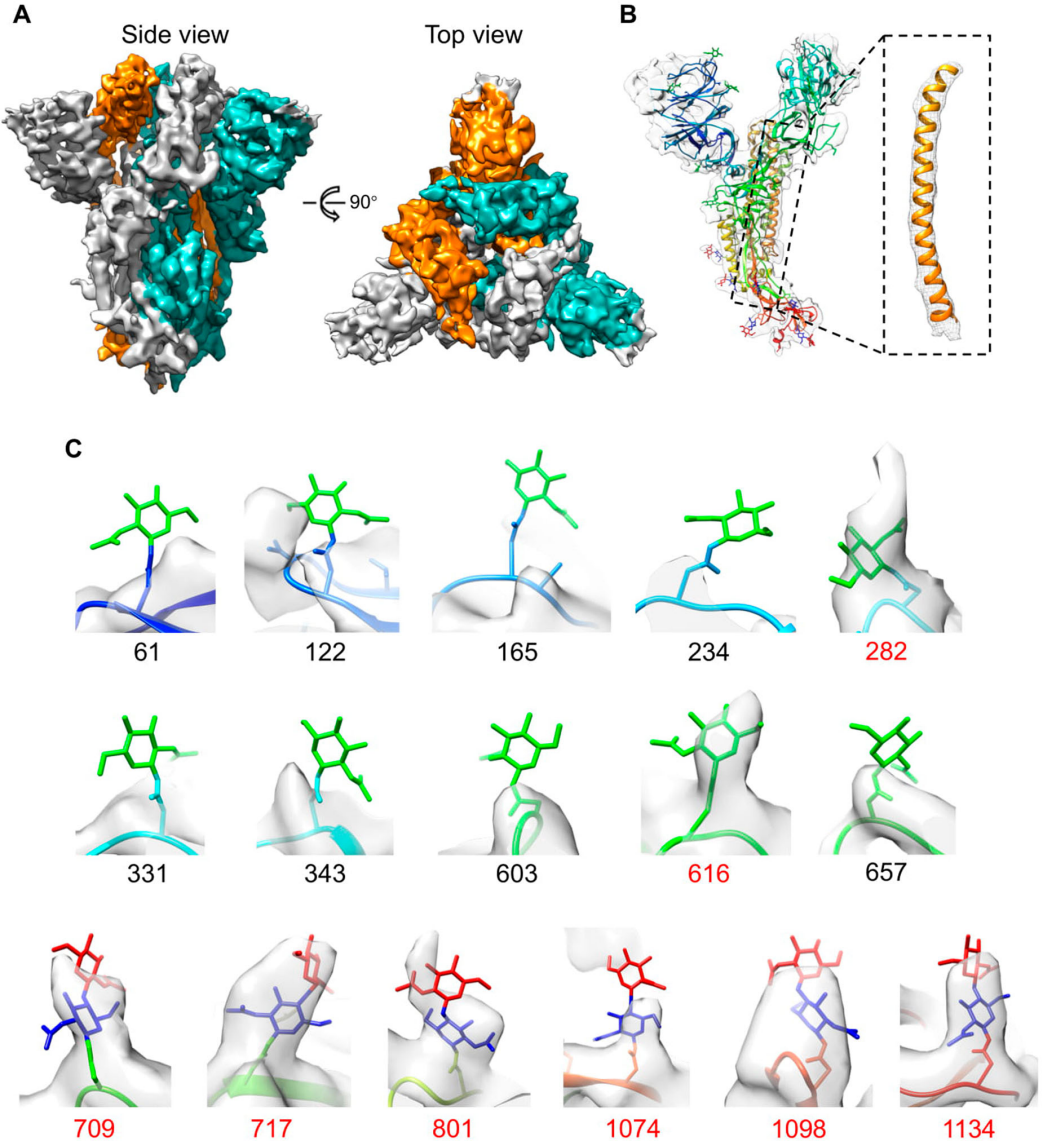
Cryo-EM structure of the trimeric S-2P and glycosylation features. (A) The 4.4-Å density map of the prefusion S trimer (S-2P), colored by protomer. (B) Model of a monomer of the prefusion S (PDB no. 6VSB, rainbow colored) was fitted into the density map of S-2P. (C) Close-up views of 16 N-linked glycan sites with decorated oligosaccharides from one S protein monomer fitted into the 4.4-Å density map. The residues with corresponding glycan densities are marked as residue number (red).

To examine the structures of the trimeric S ectodomain proteins, we prepared cryo-EM grids using Ni-NTA-purified S-WT and S-2P, and collected 1513 and 216 electron micrograph movies, respectively. For both samples, most of motion-corrected micrographs demonstrated numerous well-dispersed particles, with an approximate size of the canonical coronavirus S trimer (Supplementary Figure 1). A total of 162,645 and 19,732 particles for S-WT and S-2P, respectively, were selected for multiple rounds of 2D classification. The 2D results for both S-WT and S-2P showed typical features of an S trimer in prefusion conformation, as recently reported [9,12]. Unfortunately, 2D-classifed S-WT particles demonstrated strong orientation preferences, and none of the sorted classes assumed a side view of the trimer particles, even in the presence of detergents (e.g. DDM) or other known means of lessening orientation preference [32,33] (Supplementary Figure 1(A)). In contrast, the addition of 0.085 mM DDM gave rise to both top and side views of S-2P in 2D analysis, which allowed for subsequent 3D reconstruction (Supplementary Figure 1(B)). A total of 98,258 particles were selected for the 3D reconstruction of S-2P by applying 3-fold symmetry, yielding a density map of the prefusion spike (S-pre) at a resolution of 4.4 Å (Figure 3 and Supplementary Figure 2).

Structurally, we observe that three S monomers are intertwined around each other, associating as homotrimers, with a height of 170 Å (side-view) and diameter of 160 Å (top-view) (Figure 3(A)). We then compared the recently reported cryo-EM map of the S prefusion trimer (low-pass to 4.4 Å prior to structural comparison) with our cryo-EM map at the same resolution. Noteworthy, both trimers were engineered and expressed via the same strategy for stabilized prefusion conformation; i.e. inclusion of two stabilizing proline mutations at residues 986 and 987 and the “AGAG” substitution at the furin cleavage site [9]. Superimposition showed high similarity between these two maps (maps correlation coefficient 0.857), with a mushroom-shaped architecture (Supplementary Figure 3). Yet, the model of the prefusion S protein (PDB no. 6VSB) could be bell-fitted into our S-2P density map (average map value of model fitting: 0.542) (Figure 3(B)), indicating that the spikes in prefusion conformation from 293F mammalian cells and insect cells share identical protein folding and conformation upon trimer stabilization engineering.

A previous cryo-EM study of the 293-cell-derived spike showed that the SARS-CoV-2 S-glycoprotein comprises 22 N-linked glycosylation sequons per monomer, of which 16 of these sites bear oligosaccharides [34]. Therefore, we next checked for these 22 N-linked potential glycosylation sites in our 4.4-Å structure of S-2P (Figure 3(C)). Unexpectedly, only 8 (50%) of the 16 reported glycosylation sites were observed with discernible glycosylation in the density map of S-2P. Among these sites, glycosylation at 6 sites (N709, N717, N801, N1074, N1098, N1134) within S2 could be found in our structure, whereas only 2 (N282 and N616) of the 10 glycosylation sites within the S1 region of the 293 cell-derived spike [17] received glycosylation in the insect cell conditions. These results suggest that the S trimer expressed in insect cells presents with a different glycosylation profile than that produced in mammalian cells, particularly for the S1 subunit.

### Reactivity of SARS-CoV-2 RBD, S1, and S proteins against convalescent COVID-19 human sera

**Figure 4.**
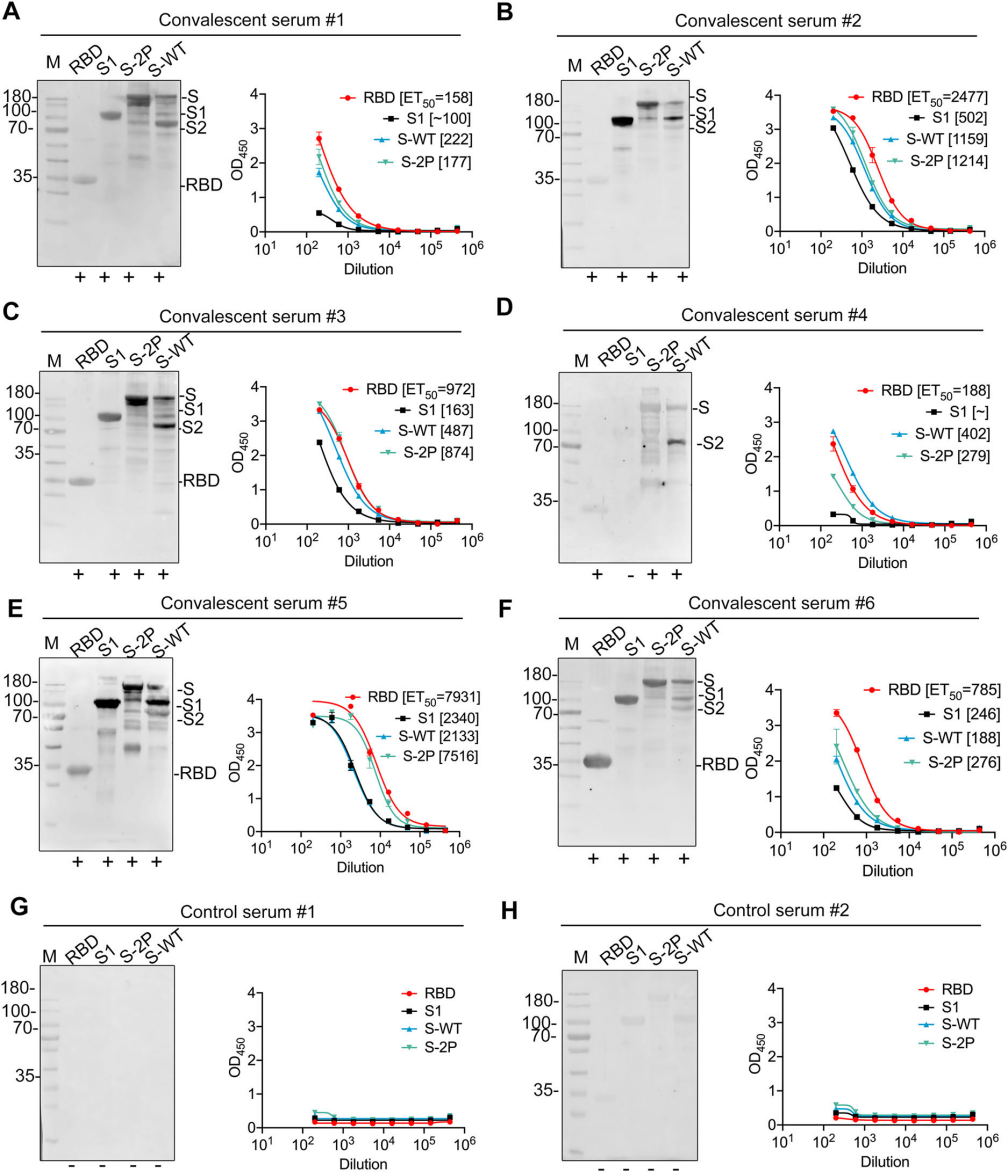
Antigenicity of the RBD, S1, and S proteins against convalescent sera. (A–F) Reactivities of the RBD, S1, S-WT, and S-2P proteins against six COVID-19 convalescent human sera (#1-#6), as detected by immunoblotting (left panel) and ELISA (Right panel). (G, H) Results of two control sera samples. The gels used for immunoblotting were duplicates of the reducing SDS gels presented in Figure 2(M).

We next evaluated the antigenicity of the four versions of the S proteins by WB and ELISA using a panel of six COVID-19 convalescent human sera, collected from COVID-19 patients after they had recovered from the disease in the First Affiliated Hospital of Xiamen University, China. Eight reducing SDS gel duplicates of the one depicted in Figure 2(M) were prepared for WB analysis using these six convalescent sera and two control sera from healthy subjects (Figure 4(A–H), left panel). As expected, the bands of S-2P and intact S-WT proteins reacted well with all six sera (Figure 4(A–H), left panel). Interestingly, four of the six sera (convalescent serum #1, #2, #3 and #5) showed weaker reactivities against the RBD as compared with the S-2P and intact S-WT proteins. Among the four sera with lower RBD-reactivity, serum samples #2 and #5 both recognized S1 and the S-WT-cleaved S1 (see lane S; Figure 4), suggesting that these sera may specifically react with the NTD of S1. S2 shared similar reactivity with S-2P and intact S-WT for all six sera. In the ELISA tests, the four proteins showed overall comparable reactivities against the six convalescent sera, except for a much lower reactivity for S1 against serum samples #1, #4, and #6. Moreover, individual serum samples showed variable reaction titres (represented as ET50) over the duration of the reactions: serum #5 > #2 > #3 > #6 > # 4 > #1 (Figure 4(A–H), right panel). No detectable reactions were observed for the control sera (Figure 4(G,H)). Overall, the four proteins exhibited good reactivities against convalescent COVID-19 patient sera in ELISA, yet showed different reaction profile for individual domain in immunoblotting.

### Immunogenicity of SARS-CoV-2 RBD, S1, S-WT and S-2P proteins in mice

**Figure 5.**
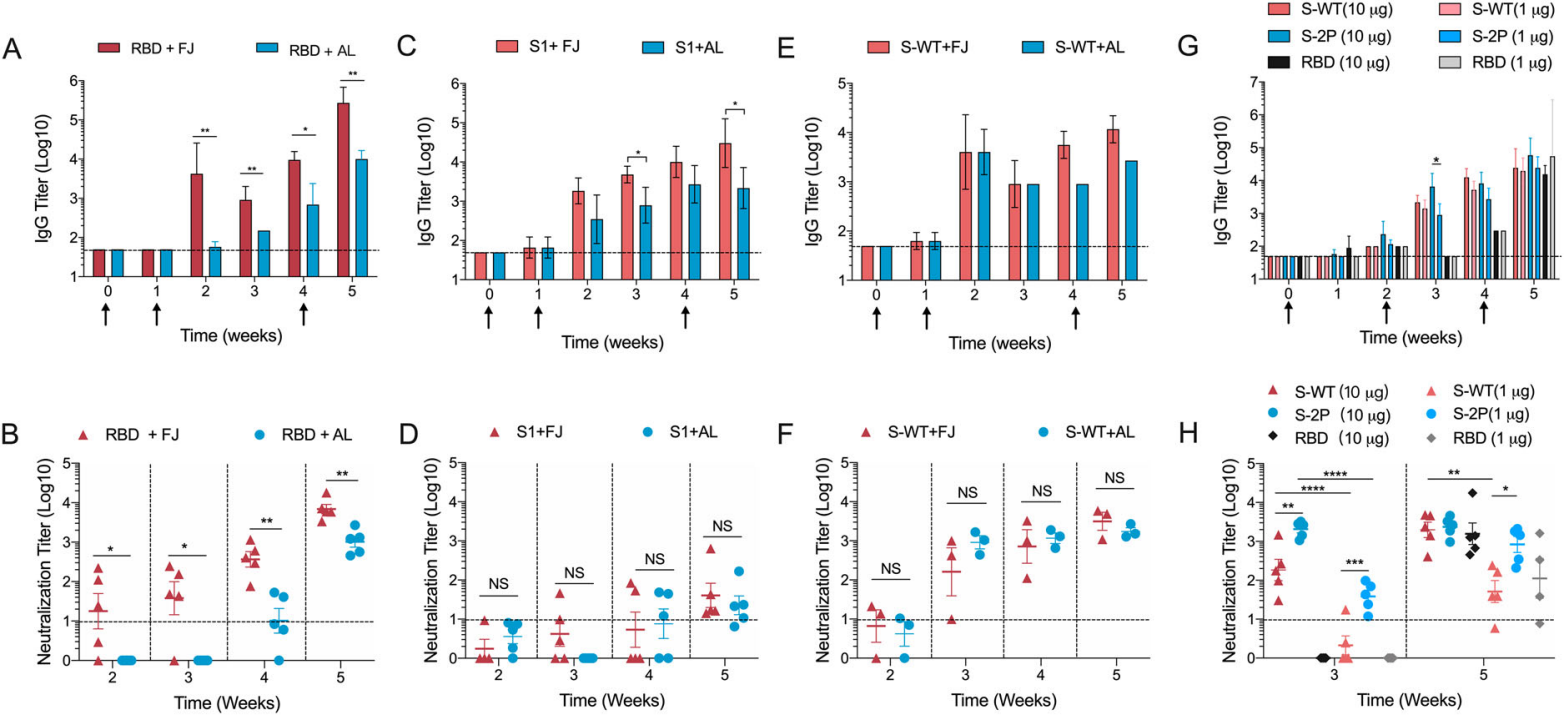
Immunogenicity of RBD, S1, S-WT, and S-2P in mice. (A, C, E) IgG titres induced by RBD, S1, and S-WT in BALB/c mice (*n* = 5). 100 µg RBD, 35 μg S1, and 35 μg S-WT were formulated with Freund’s adjuvant or aluminium adjuvant and innoculated at weeks 0, 1, and 4. (G) IgG titres induced by RBD, S-WT, and S-2P in BALB/c mice (*n* = 5). Two dosages, 1 μg and 10 μg, of each protein were formulated with aluminium adjuvant and administered at weeks 0, 2, and 4. The dotted line indicates the limit of detection for the end-point ELISA assay. (B, D, F, H) Neutralization titres of the immune sera corresponding to the samples showing IgG titres in A, C, E, and G, respectively. Graphs show the mean ± SD data in (B–F) were analyzed using unpaired *t*-tests; data in (G, H) were analyzed by two-way analysis of variance (ANOVA). *P < 0.1, **P < 0.01, ***P < 0.001, ****P < 0.0001. The neutralization titres were calculated as IC_50_ by nonlinear fit in Graphpad Prism 8.0, as shown in Supplemental Figure S1.

To evaluate the immunogenicity of these proteins, Balb/c mice (*n *= 5, per group) were administered with RBD, S1 and S-WT proteins of the SARS-CoV-2 spike (Figure 1(A)) at weeks 0, 1, and 4. Regular aluminium adjuvant or Freund’s adjuvant were used in the vaccine formulation. Antigen-specific IgG and neutralizing antibody titres were measured by end-point ELISA and cell-based vesicular stomatitis virus (VSV) pseudovirus-type neutralization assay [28], respectively. For all three proteins, the seroconversion of IgG antibodies occurred one week after the second vaccination (week 2). The Freund’s adjuvant induced significantly stronger immune response (0.5-2 log of antibody titre), for all three proteins than did the aluminium adjuvant at week 5 which was one week after the third immunization. For the RBD protein, the antibody titres with the Freund’s adjuvant were significantly higher than the titres with the aluminium adjuvant, with up to 5-log and 4-log, respectively, at week 5 (Figure 5(A)); the corresponding neutralizing antibody titres were about 4 log and 3 log, demonstrating a consistent trend in the response. In the immunization of trimeric S1 and S-WT (Figure 5(C,E)), IgG kinetic profiles were similar to that observed for the RBD groups; however, the difference between the two adjuvants was lower (less than 1 log). Unexpectedly, the neutralization titres in the S1 group were lower than those for the RBD and S-WT groups (up to 1.5 log). The S-WT elicited ∼3.5-log neutralizing antibody titres with both adjuvants (Figure 5(D,F)), indicating that S-WT is highly immunogenic for neutralizing antibody production with either of the two adjuvants.

We then compared the immunogenicity of S-WT, S-2P, and RBD at low dosages (10 μg and 1 μg) formulated with a clinically acceptable aluminium adjuvant. Mice received a three-dose immunization regime of S-WT, S-2P, or RBD at 10 μg or 1 μg dosages. The results showed that IgG seroconversion in all animals appeared at week 2 and, whereas the titres of S-WT and S-SP increased over time thereafter, the RBD titres were maintained at low levels (<2.5 log; Figure 5(G)). Intriguingly, all mice showed high IgG antibody titres (up to 4.5 log) at week 5. Of note, the IgG titres for mice administered with RBD were comparable at 1, 10 or 100 μg dosages (Figure 5(A,G)), indicating that aluminium-adjuvanted RBD is immunogenic when administered as a complete prime-boost-boost immunization regimen. In contrast, the neutralization titres for S-WT and S-2P at week 3 were dose-dependent. At week 5, 10 μg S-WT, S-2P or RBD induced comparable neutralization titres (∼3.5 log), whereas 1 μg S-2P elicited a significantly higher neutralization titre than S-WT and RBD (by ∼1 log mean value; Figure 5(H)). This suggests that the trimer-stabilized conformation of S-2P may provide higher immunogenicity at a lower immunization dosage.

### Immunogenicity of SARS-CoV-2 S-2P proteins in non-human primates

**Figure 6.**
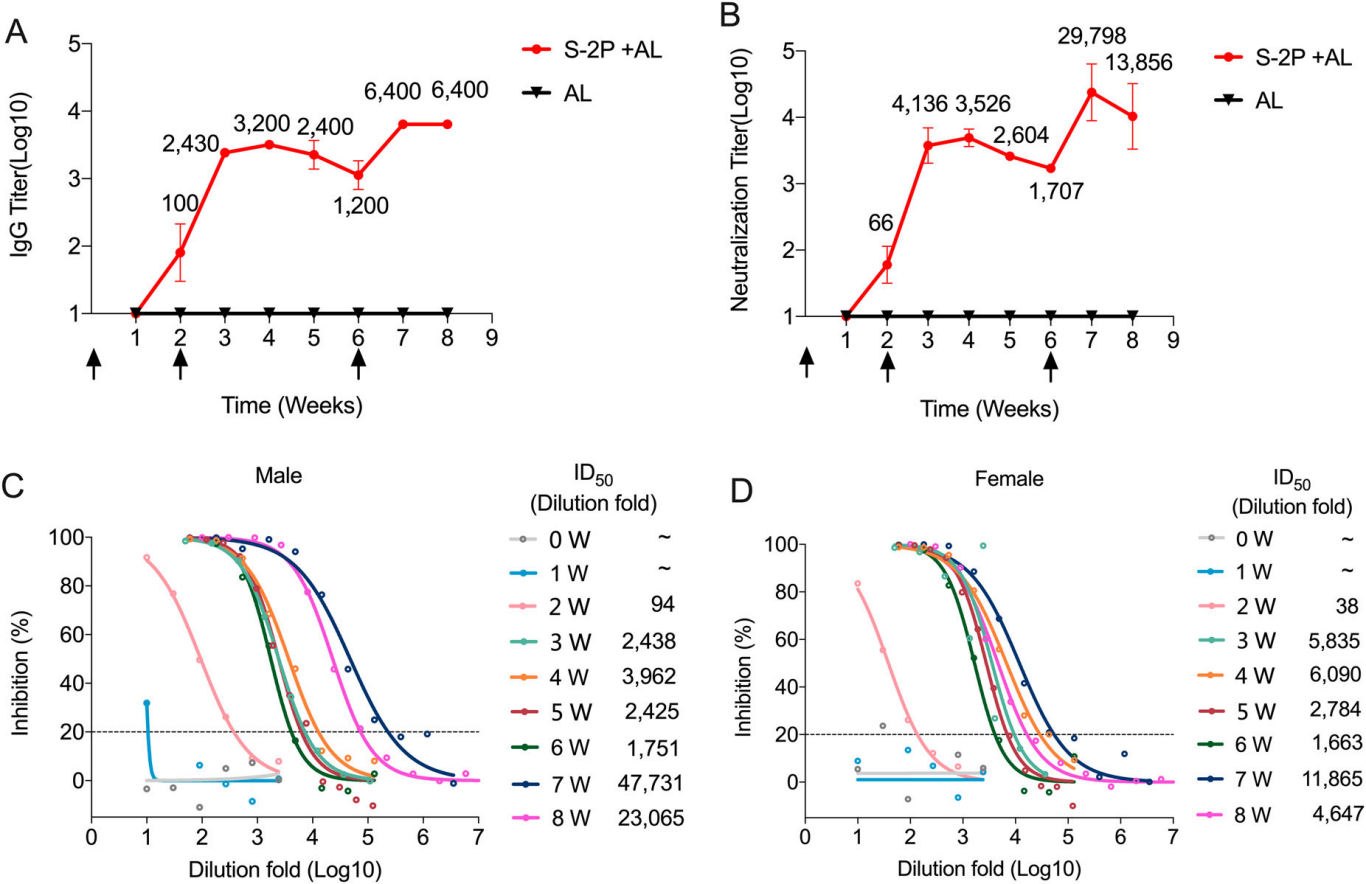
Immunogenicity of SARS-CoV-2 S-2P proteins in nonhuman primates. Cynomolgus Macaques were immunized twice at 0, 2, and 6 weeks with 20 μg of proteins or adjuvant only (*n* = 2). (A) SARS-CoV-2-specific IgG titres were measured by end-point ELISA. (B) Neutralization titres against SARS-CoV-2 were measured each week. The arrows indicate the immunization time points. (C, D) Inhibition curves of monkey sera in the neutralization assay. The mean ID_50_ (dilution fold) for two monkeys are calculated by nonlinear fit using Graphpad Prism 8. The horizontal dotted lines indicate the detection limit.

Finally, we sought to evaluate the immunogenicity of the lead S-2P immunogen in cynomolgus macaques (Macaca Fascicularis). Two test monkeys – one male and one female – were administered with a 20-μg dose of aluminium-adjuvanted S-2P at weeks 0, 2, and 6, with two control monkeys (also one male and one female) administered with aluminium adjuvant as a placebo control. The two test monkeys rendered similar kinetic responses for both SARS-CoV-2 S-specific IgG and neutralizing antibodies throughout the test period. The IgG antibody seroconverted at week 2 and reached ∼3.5 log at one week after the second vaccination (week 3), maintaining titre values of ∼3 log till week 5. After the third vaccination, the IgG antibody peaked at 3.8 log (week 7) and maintained the level at week 8 (Figure 6(A)). The neutralizing antibody response was detectable at week 2 and peaked at mean ID50 = 29,798 at week 7 (Figure 6(B–D)), consistent with the trends of IgG (Figure 6(A)). Then the mean neutralization titre slightly decreased to ID50 = 13,856 at week 8, and the neutralization titre for the male monkey was much higher than the female after the third immunization (Figure 6(C,D)). Neither the IgG nor the neutralizing antibody could be detected in the control monkeys. With respect to the neutralization titres for the panel of COVID-19 convalescent human sera (n = 18), which measured as a mean ID50 of 706 (range, 42–2698) in the exact pseudotype-based neutralization assay that showed excellent correlation with a native SARS-CoV-2 neutralization assay [28], the peak mean titre for neutralizing antibodies elicited by S-2P in the two monkeys were up to 40-times higher.

## Discussion

SARS-CoV-2 crossed the species barrier and quickly swept the globe through person-to-person transmission in a manner reminiscent of the Spanish flu pandemic of 1918. Given the rapid manifestation and spread of COVID-19, epidemiological data pertaining to the mechanism of SARS-CoV-2 infection is still in its infancy, and an efficacious vaccine that can control or even eliminate the virus is still wanting. Building on the knowledge garnered from the other recent SARS- or MERS-CoV outbreaks, we know that the spikes decorating the SARS-CoV-2 virion play a critical role in viral attachment and entry into host cells and are thus also likely to be the major target for vaccine design. Consequently, numerous SARS-CoV-2 vaccine candidates, including inactivated, vectored, recombinant and nucleotide vaccine forms, are currently being tested in clinical trials or are at other stages of preclinical research [35].

Native-like spike trimers of SARS- or MERS-CoV have been successfully generated in various eukaryotic expression systems, such as CHO, 293, and insect cells, and their use in the immunization of mice and hamsters [29,36–38] has shown efficacy in disease protection. Among these systems, insect cells are phylogenetically distant to human cells, and bear a similar yet simpler glycosylation pattern that is established during post-translational modification [39]. Furthermore, insect cells are a well-established expression system that is easy to scale-up and usually results in high productivity. The utility of the insect cell system is exemplified by the recombinant human influenza vaccine (FluBlok®), the virus-like particles (VLP) vaccine targeting papillomavirus (Cervarix®), and the therapeutic cancer vaccine, Provenge®. Here, we show that the RBD, NTD, and S2 domains of SARS-CoV-2 are glycosylated to different extents by the insect cell system, with a generally lower level of glycosylation (extent and sites) as compared with that derived from a mammalian expression system. Using PNGase F, we measured only a 20-kD reduction in the molecular weight of the S protein through glycan removal as compared with the approximately 190-kD loss that has been measured for S proteins produced in mammalian expression systems [34]. For the S-WT proteins, S1 and S2 were partially cleaved, with their positions on immunoblots indicating that the S2 protein has a heavier degree of glycosylation than the S1 protein (Figure 2(N)). In vaccine development for other enveloped viruses, the extent of glycosylation on immunogens can have a profound effect on the level of the immune response generate; indeed, in some cases, more complex and larger glycans linked to envelop proteins will mask immunogenic epitopes, with prior deglycosylation or reducing the length of the glycans able to improve the immunogenicity. For example, influenza virus design was improved by deglycosylation [40], HIV by selectively removing the glycans [41], and HCV by expression in insect cells [42]. In this study, the S protein from insect cells showed much more glycosylation on the S2 subunit than on the S1 subunit. This information should be considered in SARS-CoV-2 vaccine design, because the S1 subunit contains the RBD, which harbours a considerable number of potent, conformation-dependent epitopes that are associated with hACE2 receptor binding. The reduced glycosylation levels from its production in insect cells as compared with mammalian cells may affect its binding potency. Similarly, the S2 subunit is relatively conserved in terms of its sequence across different virus isolates [43]; whether the higher levels of glycosylation on the S2 subunit in insect cells will affect its function will also require further study.

On the aspect of antigenicity, the RBD, S1 and S proteins reacted well with the convalescent sera, suggesting that these proteins maintain the native-like SARS-CoV-2 epitopes that are immunogenic in COVID-19 patients. The western blotting results further demonstrated that most of the RBD epitopes are relatively tertiary conformation-dependent, which could be damaged under mild denaturation conditions (reducing conditions and SDS treatment), and that the NTD within the S1 subunit bears some linear epitopes, whereas the S2 region essentially has linear epitopes.

In the immunogenicity assay, S1 induced very low neutralization titres even when administered with Freund’s adjuvant, and this was reflected by the lower reactivity of S1 against human sera. However, both RBD and the two forms of the S trimers, while formulated with Freund’s and aluminium adjuvants, could elicit considerable neutralizing antibody levels with a dose as low as 1 µg. It is worthy noted that the neutralization titres elicited by 10 μg and 100 μg aluminium adjuvanted RBD were comparable in mice, which might be due to a two-fold reason, (1) immunization programme of 0, 2, 4w may facilitate the antibody response in 10 μg group (Figure 5(H)) rather than 0, 1, 4w in 100 μg dose, which might be associated with a longer B cell maturation timer [44] in 0, 2, 4w regiment; (2) The dose dependency of RBD immunization with 3-dose administration may reach saturated at about 10 μg, demonstrating comparable neutralization titres at 10 μg and 100 μg, but significantly lower at 1 μg. We have found this phenomenon in immunogenicity data for the chimeric HPV33/52/58 VLPs, where 10 μg and 1 μg induced comparable neutralization titres but significantly lower for 0.1 μg [45]. A recently reported inactivated vaccine candidate (PiCoVacc) was able to induce SARS-CoV-2 neutralization titres in rhesus macaques that were comparable with the convalescent sera from recovered COVID-19 patients, but still conferred efficacious protection against SARS-CoV-2 challenge [19]. In addition, a DNA vaccine encoding the full-length S protein produced neutralizing antibody titres associated with vaccine protective efficacy in rhesus macaques, with the neutralization titres exhibiting comparable magnitudes to the mean neutralizing antibody titres from a cohort of convalescent humans (*n* = 27) [46]. By comparison, vaccination with S-2P at the 20-μg dose in cynomolgus macaques in our study elicited high IgG titres and excellent mean neutralization titres, reaching 4-log values at one week after the third immunization; this is estimated to be about 40 times that of convalescent human sera (*n* = 18) [28].

The spike proteins decorated on the SARS-CoV-2 surface meditate the virus entry via two conformational states, prefusion one is metastable and responsible for ACE2 receptor recognition at the first stage, whereas post-fusion state conformationally transformed from prefusion one enables the eventual virus-cell membrane fusion for virus penetration into host cells, which is suggested by the evidences found on the cognate SARS-CoV [47] and MERS-CoV [48]. In terms of the role of prefusion associated with the first stage of virus infection, vaccine design by stabilizing the prefusion states has been underscored not only in other coronaviruses but also in other membrane viruses such as RSV [49,50] and HIV [51], which was usually materialized by structure-guided introduce of relatively rigid proline residue, disulfide bonds or space-filling substitutions. The stabilized prefusion proteins, mostly in trimeric form, were verified to preserve those native epitopes reciprocal to receptor binding sites and therefore could elicit high-quality neutralizing antibodies against virus infection rather than unmutated S proteins. In this work, we constructed the SARS-CoV-2 S-2P at the early stage of COVID-19 outbreak by learning the lessons of SARS-CoV and MERS-CoV spike, where furin cleavage site was silent and the prefusion conformation was stabilized by two prolines [29]. Our results also demonstrated that the neutralizing titre elicited by S-2P is higher than S-WT with partial furin cleavage. This S-2P design has been adopted in other cases of the vaccine development including NVX-CoV2373 [52] and mRNA-1273 [53] that are in clinical trials. Whether the high neutralization titre by the recombinant S-2P proteins based on BEVS could translate into spectacular efficacy against COVID-19 and even SARS-CoV-2 infection remains to be determined in animal models, such as hACE2 transgenic mouse [54], hamsters [55], and monkeys [19]. Nonetheless, the prefusion trimer-stabilized S-2P despite of in non-particulate form seems to afford higher neutralization titres than other reported vaccine candidates, and thus holds promise for the development of a recombinant vaccine against SARS-CoV-2.

## Supplementary Material

BacS_manuscript_SI_EMI_July-16th.docx

## Data Availability

EM map of the S-2P trimer is available at the Electron Microscopy Data Bank (EMDB), under the accession no. EMBD-30506. All other data to support the conclusions are in the main paper or supplementary materials.
