# Supplementary material for: SARS-CoV-2 spike produced in insect cells elicits high neutralization titres in non-human primates
Source: Emerg Microbes Infect. 2020 Sep 24;9(1):2076–90. doi: 10.1080/22221751.2020.1821583 (PMC7534368; doi:10.1080/22221751.2020.1821583)
Supplement: BacS_manuscript_SI_EMI_July-16th.docx [file TEMI_A_1821583_SM9141.docx]

Supplementary Materials for

Title: SARS-CoV-2 spike produced in insect cells elicits high neutralization titers in non-human primates

Authors: Tingting Li^1,2,#^, Qingbing Zheng^1,2,#^, Hai Yu^1,2,#^, Dinghui Wu^3,#^, Wenhui Xue^1,2,#^, Hualong Xiong^1,2,#^, Xiaofen Huang^1,2^ , Meifeng Nie^1,2^, Mingxi Yue^1,2^, Rui Rong^1,2^, Sibo Zhang^1,2^, Yuyun Zhang^1,2^, Yangtao Wu^1,2^, Shaojuan Wang^1,2^, Zhenghui Zha^1,2^, Tingting Chen^1,2^, Tingting Deng^1,2^, Yingbin Wang^1,2^, Tianying Zhang^1,2^, Yixin Chen^1,2^, Quan Yuan^1,2^, Qinjian Zhao^1,2^, Jun Zhang^1,2^, Ying Gu^1,2,*^, Shaowei Li^1,2,*^, Ningshao Xia^1,2,4*^

Correspondence to: [nsxia@xmu.edu.cn](mailto:nsxia@xmu.edu.cn) (N.X.); [shaowei@xmu.edu.cn](mailto:shaowei@xmu.edu.cn) (S.L.); [guying@xmu.edu.cn](mailto:guying@xmu.edu.cn) (Y.G.)

**This PDF file includes:**

Figs. S1 to S4

Fig. S1. Cryo-EM raw images (left) and 2D classification analysis (right) of S-WT (A) and S-2P (B). Both samples were incubated in 0.085 mM dodecyl-maltoside (DDM). The red dashed boxes in (B) indicate particles in sideviews.

**Fig. S2. Fourier shell correlation curve for 3D reconstruction of S-2P.**

**Fig. S3. Cryo-EM structural comparisons of 2-2P and reported prefusion S trimer.** A. the 4.4 Å cryo-EM map of S-2P. B. the reported prefusion S trimer (EMD-21374) which was low-passed to 4.4Å. C. Alignment of above two maps show high structure similarity (correlation coefficient 0.857).


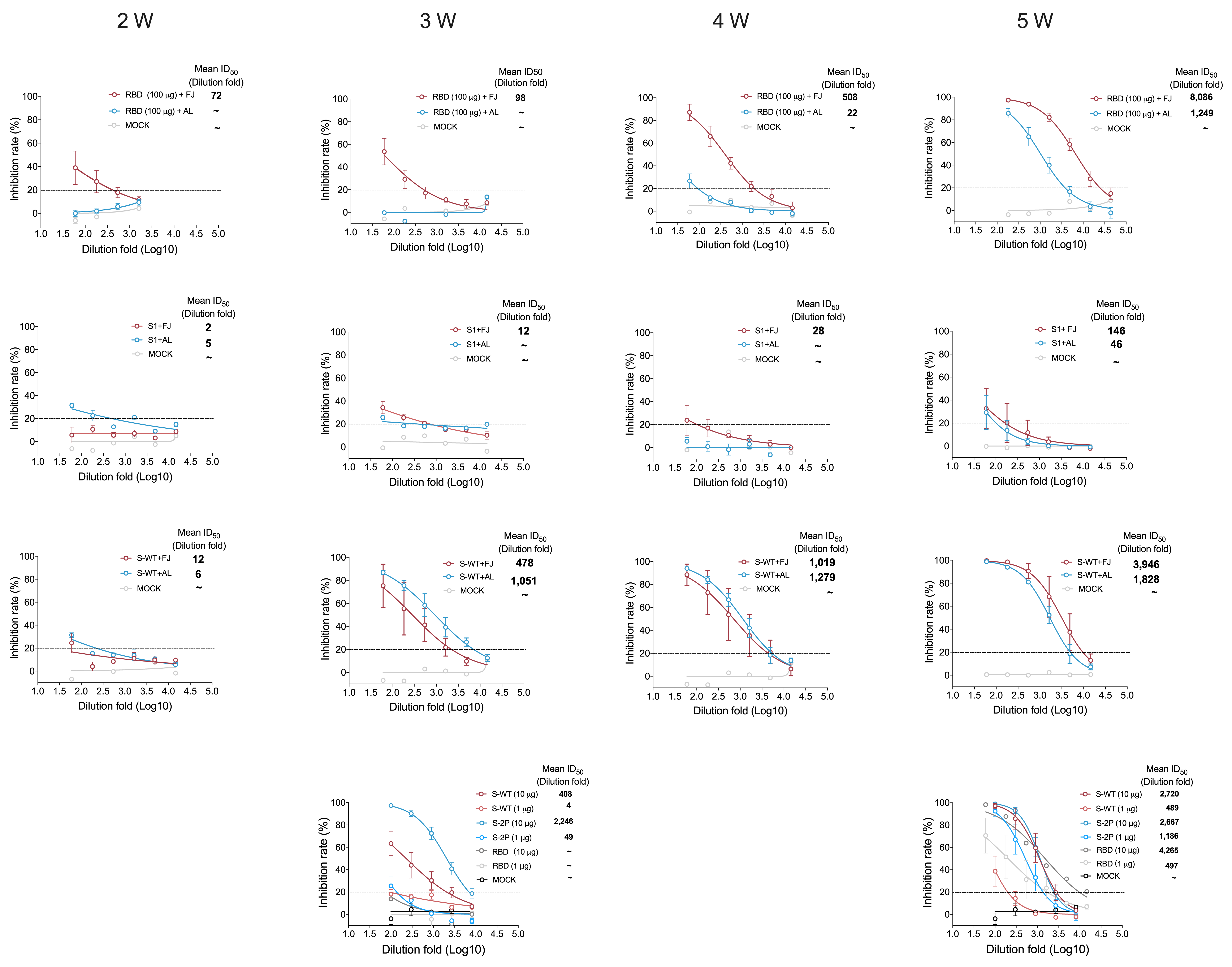


**Fig. S4. Neutralization titers of the sera of mice.** The curve was fitted by the inhibition response model and the ID_50_ was calculated by Graphpad Prism 8. Dotted lines indicate the limit of detection. The Mean ID_50_ was obtained by averaging the ID_50_ values of individual mice.
